# Evaluating the repeatability of corneal elevation through calculating the misalignment between Successive topography measurements during the follow up of LASIK

**DOI:** 10.1038/s41598-017-03223-9

**Published:** 2017-06-09

**Authors:** XiaoBo Zheng, WeiHua Yang, LiFang Huang, JunJie Wang, Si Cao, Brendan Geraghty, YiPing Zhao, QinMei Wang, FangJun Bao, Ahmed Elsheikh

**Affiliations:** 1 The Affiliated Eye Hospital of Wenzhou Medical University, Wenzhou, 325027 China; 2 The institution of ocular biomechanics, Wenzhou Medical University, Wenzhou, Zhejiang Province 325027 China; 3 The first Affiliated Hospital of Huzhou University, Huzhou, Zhejiang 313000 China; 4 School of Engineering, University of Liverpool, Liverpool, L69 3GH UK

## Abstract

The study aims to evaluate, using the Iterative Closest Point (ICP) algorithm, the repeatability of successive corneal elevation measurements taken post-LASIK. Two topography maps of 98 LASIK participants were recorded preoperatively (Pre), 1 month (Pos1M) and 3 months postoperatively (Pos3M). Elevation of the second measurement was fitted to the first measurement by calculating using ICP, and correcting for, both translational and rotational misalignment components. The RMS of elevation differences between anterior corneal measurements were statistically significant post-LASIK compared to preoperation (P < 0.05). A misalignment ratio used to describe the weighting of the elevation difference caused by misalignment relative to the total difference remained stable (0.40 and 0.23 for anterior and posterior corneal surfaces, respectively) in different periods. The study also considered the combined misalignment parameter (CM), which represents the total effect of all individual misalignment components on the repeatability of corneal topography maps. CM was significantly greater post-LASIK relative to pre-LASIK (P < 0.05). Overall, the contribution of misalignment to the total difference between successive corneal measurements remained stable pre and post operation, while the combined effect of refractive error correction and optical diameter appeared to have a significant influence on the elevation repeatability in the early stages of the follow up period.

## Introduction

The cornea contributes approximately two thirds to the total refractive power of the eye^1^. Recent advances in technology to measure corneal topography are allowing clinicians to better evaluate the cornea and its continuation to ocular refractive power^2^. Reliable characterisation of corneal topography is critical for the evaluation of visual quality and in several applications including, the planning of refractive surgery^3, 4^, diagnosis and management of corneal disorders^5, 6^, and contact lens fitting^7, 8^. For these applications and for the longitudinal monitoring of progressive corneal conditions, corneal topography measurements available to clinicians should be repeatable.

While Placido disc–based reflective topography can only map the anterior surface of the cornea^2^. Scheimpflug tomography can measure the elevation data (height) of both the anterior and posterior corneal surfaces. Compared with Placido disc-based systems, the corneal elevation profile can represent the shape of corneal surface more accurately^9^, and locate the corneal apex post laser *in situ* keratomileusis (LASIK) more efficiently. Consequently, the Pentacam (which employs Scheimpflug technology) has become a valuable tool for corneal anterior segment analysis, and known for its repeatable anterior and posterior corneal measurements^10–12^. Earlier studies also showed evidence the Pentacam provides repeatable measurements of biometric parameters such as corneal curvature, central corneal thickness and anterior chamber depth (ACD)^12–16^ in normal corneas.

Keratorefractive surgery, particularly LASIK, has become a commonly used procedure for the correction of myopia. In order to correctly assess the outcome of LASIK, it is important for clinicians to accurately determine corneal topography postoperatively. Erroneous postoperative corneal topography measurements may mislead the clinical decision regarding corneal surgery enhancement^17^, estimation of intraocular lens power^4, 18^ and measurement of the intraocular pressure^19^. In these applications, the high repeatability of topography measurements is essential for ensuring reliability in monitoring changes in corneal shape during surgery follow-up.

Repeatability, or the consistency between readings for the same subject taken on the same instrument under constrained conditions, should be high. Data on repeatability of different videokeratography instruments can be found in the literature^16, 20^; however, data regarding the repeatability of measurements from a global perspective is scarce. Reliance on point matching methods in earlier studies on three-dimensional (3-D) topography testing is considered a limitation, in which discrete points located in different regions of corneal surface should be picked up to get an overall impression of the topography measurement. Therefore, the present study is designed to compare the repeatability of Pentacam measurements in pre-operative, 1 month and 3 months post-operative eyes using a surface matching method can estimate and correct for the misalignment between successive topography measurements.

## Results

The surgery parameters for the 98 eyes included in the study included the optical diameter (OD = 6.58 ± 0.34 mm), residual stromal bed thickness (RSB = 342.09 ± 26.07 μm), and maximum ablation depth (MAD = 95.68 ± 21.62 μm). The measurement results of corneal curvature in the horizontal (K_h_) and vertical (K _v_) directions, the corneal shaper factor in 30 degree (Q_30_), and central corneal thickness (CCT) are shown in Table 1. Although K_h_, K _v_ and CCT decreased and Q_30_ increased postoperatively than in preoperation (P < 0.05, Table 1), there were no significant differences between successive measurements of these four biometric parameters taken in 3 different periods (Pre, Pos1M and Pos3M) (P > 0.05, Table 1).

**Table 1:** Mean and inter-measurement difference of conreal biometric parameters between two successive measurements among three periods.

Periods	K_h_, D		K _v_, D		Q_30_		CCT, μm	
	Mean	Diff	Mean	Diff	Mean	Diff	Mean	Diff
Pre	42.73 ± 1.38	−0.01 ± 0.15	43.90 ± 1.67	0.03 ± 0.18	−0.27 ± 0.11	0.00 ± 0.06	543.27 ± 24.85	0.04 ± 4.45
Pos1M	38.04 ± 1.97	−0.01 ± 0.22	38.49 ± 2.13	−0.01 ± 0.31	0.40 ± 0.5	0.02 ± 0.14	446.07 ± 30.1	0.49 ± 4.84
Pos3M	38.74 ± 1.97	0.07 ± 0.27	39.25 ± 2.05	−0.01 ± 0.36	0.39 ± 0.43	0.02 ± 0.09	451.56 ± 32.4	1.25 ± 4.23
Pre vs Pos1M	0.00**	1.00	0.00**	0.66	0.00**	0.42	0.00**	0.81
Pre vs Pos3M	0.00**	0.06	0.00**	0.61	0.00**	0.45	0.00**	0.23
Pos1M vs Pos3M	0.05	0.10	0.07	0.99	0.97	0.99	0.50	0.62

Pre means the period pre operation, Pos1M means 1 month post LASIK, Pos3M means 3 months post LASIK. Mean represents the average of the first and second measurements result, while Diff represents the inter-measurement-difference of two successive measurments (second mesurement – first mesurement), *means P < 0.05, **means P < 0.01.

Topography match results for the 98 subjects can be seen in Tables 2, 3, 4, 5 and 6. For the anterior corneal surface, the mean differences between successive elevation maps (calculated both before and after misalignment correction using ICP; PreICP-RMS and PosICP-RMS) were significantly higher one month after surgery (Pos1M) and three months after surgery (Pos3M) compared to before surgery (Pre). On the other hand, the misalignment ratio, which describes the weighting of difference between successive measurements caused by misalignment to the total difference remained similar at approximately 0.40 (Tables 2 and 3) in different periods (Pre, Pos1M and Pos3M). For the posterior corneal surface, only PreICP-RMS at Pos3M was statistically significant when compared to Pre. Further, the mean differences between elevation maps (calculated both before and after misalignment correction using ICP; PreICP-RMS and PosICP-RMS) were not statistically significant one month after surgery (Pos1M) compared to both before surgery (Pre) and three months after surgery (Pos3M). The misalignment ratio in posterior corneal surface data at all stages remained constant at approximately 0.23 (Tables 2 and 3).

**Table 2:** Area and matching error results of the the first and second measurement.

Periods	Corneal surface	PreICP-RMS, μm	PosICP-RMS, μm	Misalignment ratio	PreICP-Area, mm^2^	PosICP-Area mm^2^	Overlap Area Ratio
Pre	Anterior	5.66 ± 3.96	3.08 ± 2.14	0.40 ± 0.21	86.34 ± 7.42	84.56 ± 7.47	0.979 ± 0.006
	Posterior	15.37 ± 8.45	12.02 ± 7.04	0.20 ± 0.18	70.80 ± 5.89	69.25 ± 5.93	0.978 ± 0.006
Pos1M	Anterior	9.33 ± 7.83	5.18 ± 4.08	0.39 ± 0.2	83.46 ± 8.68	81.56 ± 8.89	0.977 ± 0.010
	Posterior	18.15 ± 12.32	13.25 ± 10.18	0.25 ± 0.19	69.08 ± 6.97	67.42 ± 7.15	0.976 ± 0.009
Pos3M	Anterior	8.69 ± 5.96	4.59 ± 2.2	0.40 ± 0.2	82.66 ± 9.77	80.76 ± 9.9	0.977 ± 0.008
	Posterior	20.58 ± 13.44	15.49 ± 11.76	0.24 ± 0.18	68.21 ± 7.84	66.55 ± 7.93	0.975 ± 0.008

Pre means the period pre operation, Pos1M means 1 month post LASIK, Pos3M means 3 months post LASIK; PreICP-RMS and PosICP-RMS represent the root-mean-square error of the coordinate differences of corneal surface between two successive measurements before and after topography matching, respectively; Misalignment ratio = 1- PosICP-RMS/PreICP-RMS; PreICP-Area, PosICP-Area represent the Overlap area of corneal surface between two successive measurements before and after topography matching, respectively; Overlap Area Ratio = Pri-Area/Cor-Area.

**Table 3:** Comparison of area and matching error results of the the first and second measurement.

Comparison	P						
	Corneal surface	PreICP-RMS, μm	PosICP-RMS, μm	Misalignment ratio	PreICP-Area, mm^2^	PosICP-Area mm^2^	Overlap Area Ratio
Pre vs Pos1M	Anterior	0.000**	0.000**	1.000	0.115	0.101	0.154
	Posterior	0.381	1.000	0.210	0.365	0.315	0.134
Pre vs Pos3M	Anterior	0.005**	0.004**	1.000	0.027*	0.023*	0.092
	Posterior	0.014**	0.078	0.589	0.063	0.054	0.087
Pos1M vs Pos3M	Anterior	1.000	0.763	1.000	1.000	1.000	1.000
	Posterior	0.703	0.584	1.000	1.000	1.000	1.000

Pre means the period pre operation, Pos1M means 1 month post LASIK, Pos3M means 3 months post LASIK; PreICP-RMS and PosICP-RMS represent the root-mean-square error of the coordinate differences of corneal surface between two successive measurement before and after topography matching, respectively, Misalignment ratio = 1 - PosICP-RMS/PreICP-RMS; PreICP-Area, PosICP-Area represent the Overlap area of corneal surface between two successive measurements before and after topography matching, respectively; Overlap Area ratio = Pri-Area/Cor-Area; *means P < 0.05, **means P < 0.01.

**Table 4:** Translational and rotational displacement results of the second measurement compared to the first one.

Periods	α, degree	β, degree	γ, degree	x_0_, μm	y_0_, μm	z_0_, μm	CM, μm
Pre	−0.15 ± 0.63	0.11 ± 0.51	−0.1 ± 2.35	14.56 ± 70.24	20.52 ± 86.76	−0.68 ± 2.1	6.69 ± 5.01
Pos1M	0.01 ± 0.55	−0.05 ± 0.56	0.49 ± 3.71	−5.75 ± 85.62	0.55 ± 84.07	−0.83 ± 1.71	10.49 ± 8.73
Pos3M	−0.03 ± 0.49	0.11 ± 0.56	−0.63 ± 3.77	18.11 ± 85.36	2.71 ± 74.19	−0.4 ± 1.57	9.85 ± 7.74
Pre vs Pos1M	0.277	0.199	0.772	0.354	0.428	1.000	0.003**
Pre vs Pos3M	0.555	1.000	0.935	1.000	0.579	1.000	0.019*
Pos1M vs Pos3M	1.000	0.270	0.166	0.302	1.000	0.642	1.000

Pre means the period pre operation, Pos1M means 1 month post LASIK, Pos3M means 3 months post LASIK; α, β, γ represent the angular rotations about the three main axes (x, y and z) of anterior and posterior corneal surface, respectively; x_0_, y_0_, z_0_ represent the translational displacements of anterior and posterior corneal surface, respectively; Combined misalignment parameter (CM) developed to combine the effect of all these six misalignment components.

**Table 5:** The correlation result of PreICP-RMS and PosICP-RMS for anterior and posterior corneal surface with surgery parameters and SE in different periods post LASIK (Pos1M and Pos3M).

Comparison	Corneal surface	Pos1M		Pos3M	
		PreICP-RMS, μm	PosICP- RMS, μm	PreICP -RMS, μm	PosICP-RMS, μm
OD	Anterior	−0.469**	−0.312*	−0.302*	−0.358**
	Posterior	−0.223	−0.122	−0.174	−0.200
RSB	Anterior	−0.355*	−0.379*	−0.248	−0.267
	Posterior	−0.114	−0.172	0.174	0.184
MAD	Anterior	0.350*	0.363*	0.266	0.313*
	Posterior	0.269	0.250	0.024	−0.074
SE	Anterior	−0.331*	−0.200	−0.277*	−0.335*
	Posterior	−0.174	−0.183	−0.099	−0.089

Pos1M means 1 month post LASIK, Pos3M means 3 months post LASIK; PreICP-RMS and PosICP- RMS represent the root-mean-square error of the coordinate differences of corneal surface between two successive measurement before and after topography matching, respectively; OD represents optical diameter, RSB means residual stromal bed thickness MAD was maximum ablation depth and SE was spherical equivalent.

**Table 6:** The correlation result of PreICP-RMS and PosICP-RMS for anterior and posterior corneal surface with surgery parameters after correction of SE in different periods post LASIK (Pos1M and Pos3M).

Comparison	Corneal surface	Pos1M		Pos3M	
		PreICP-RMS, μm	PosICP-RMS, μm	PreICP-RMS, μm	PosICP-RMS, μm
OD	Anterior	−0.084	0.022	−0.017	−0.048
	Posterior	−0.035	0.064	−0.083	−0.106
RSB	Anterior	−0.074	−0.162	−0.112	−0.113
	Posterior	0.152	0.160	0.299*	0.238
MAD	Anterior	−0.186	0.121	−0.039	−0.055
	Posterior	−0.179	−0.067	−0.208	−0.273

Pos1M means 1 month post LASIK, Pos3M means 3 months post LASIK; PreICP-RMS and PosICP-RMS represent the root-mean-square error of the coordinate differences of corneal surface between two successive measurement before and after topography matching, respectively; OD represents optical diameter, RSB means residual stromal bed thickness, MAD was maximum ablation depth and SE was spherical equivalent.

The area of overlap from the topography matching of successive measurements decreased from Pre to Pos3M, but not between Pre and Pos1M or between Pos1M and Pos3M (Table 3). Most of the rotational and translational misalignment parameters in the Pre stage were different from zero (p < 0.05) except for γ (p = 0.67). However, this was not the case at the Pos1M and Pos3M stages where most of the rotational and translational misalignment parameters were not different from zero (p > 0.05) except for z_0_ (p < 0.00). Although some misalignment parameters were different from zero, the differences were too small to be considered important in clinical practice. All of the rotational and translational misalignments parameters (x_0_, y_0_, z_0_, α, β, γ) between successive measurements in different periods (Pre, Pos1M and Pos3M) were not significant when compared within each stage (Pre vs Pos1M, Pre vs Pos3M, Pos1M vs Pos3M, P > 0.05) (Table 4). Further, the combined misalignment parameter (CM), which aims to combine the effect of all misalignment components on topography misalignment, was significantly lower in the Pre stage than it was at the Pos1M and Pos3M stages (Table 4).

The correlation of PreICP-RMS and PosICP-RMS for both anterior and posterior surfaces with the surgerical parameters and with the spherical equivalent error (SE) in different postoperative periods (Pos1M and Pos3M) are concluded in Table 5. All the PreICP-RMS and PosICP-RMS for the anterior surface were correlated with surgerical parameters and SE in Pos1M, while the correlation decreased in Pos3M. For the posterior surface, all correlations decreased when compared to the anterior surface and none of them was significant. The correlations of OD with PreICP-RMS and PosICP-RMS was much higher than RSB, MAD and SE. OD was statistically correlated with SE (r = 0.688, P = 0.00). However, after correcting for the effects of SE, all correlations decreased and most of them became insignificant (Table 6).

## Discussion

The Pentacam utilises a Scheimpflug camera and a monochromatic slit light source, which rotate together around the ocular optical axes to acquire images of the anterior and posterior surfaces of cornea and offer a non-invasive method of obtaining a three-dimensional representation of the anterior segment. Corneal elevation, according to which corneal curvature and pachymetry are calculated, is the primary data obtained by Pentacam. In order to carry out a repeatability analysis of corneal biometric parameters, a large number of points located in the different regions are required to make a comprehensive evaluation^20–22^. In these studies, the repeatability analysis results from different regions are separated and would not provide an overall impression of the whole corneal surface. The present study builds on earlier repeatability assessments and aims to concentrate on the repeatability of the Pentacam in eyes undergoing LASIK surgery.

A common feature of most of the current videokeratography systems is that they are viewer-centred and so their accuracy and reliability will be influenced by fixation lags and eye movements. Topography devices will reject large misalignments or compensate for them during data acquisition^13, 23^. However, smaller misalignments remain and may be unavoidable. Misalignments of corneal topography measurements can affect estimates of corneal biometric parameters such as corneal curvature, corneal asphericity and corneal thickness.

Overall, the Pentacam measurements in post-LASIK eyes have two possible sources of errors, systematic and random^24^, and it is difficult to separate their effects. Systematic errors include reduced accuracy in peripheral and posterior corneal regions, and optical distortion caused by aberrations in the Pentacam’s measuring lens, both leading to differences between successive topography measurements. On the other hand, random errors may be caused by an altered corneal refractive index, mistaken detection of stromal interface, variation in magnification ratio of the posterior cornea, stromal haze, change in transparency in the early postoperative cornea, and alterations in shape reconstruction algorithms^25–28^. These random errors may alter corneal shape measurements, causing further differences between successive maps and running risks of inappropriate retreatment decisions or misdiagnosis of early iatrogenic keratectasia after LASIK. In this study, the translational and rotational misalignments (x_0_, y_0_, z_0_, α, β, γ) between successive measurements are considered an important, albeit not the only, cause of random errors, and quantified herein when assessing the repeatability of topography maps.

In the post-LASIK stage, the surface analysis was more complex when compared with the more spherical corneas encountered in the pre-operation stage due to the central flattening caused by excimer ablation. The elevation differences (PreICP-RMS) between two succesive measurements increased significantly post-LASIK when compared with pre-operation. The result was similar to previous studies where the repeatability of the Pentacam decreased in eyes with corneal thinning and contour changes after refractive surgery^29^. However, the significant difference in elevation data should be considered alongside the non-significant differences observed in the corneal biometric parameters obtained before and after LASIK (Table 1).

LASIK corrects refractive errors by reshaping the anterior corneal surface. In the present study, the PreICP-RMS and PosICP-RMS for the anterior corneal surface were correlated with surgerical parameters in Pos1M and Pos3M, but not for the posterior surface (Table 5). The results agreed with the fact that changes in anterior corneal surface (direct surgery effects because of laser ablation) were greater than those in the posterior surface (indirect effects caused by surgical biomechanical effect).

The results further show that the correlations of OD with PreICP-RMS and PosICP-RMS were stronger than for RSB, MAD and SE. However, most correlations of PreICP-RMS and PosICP-RMS with OD, RSB and MAD became non-significant after correcting for the effects of SE. MAD, which is dependent on myopic diopter correction and optic zone diameter, is nomally restricted to be ≤130 μm when considering the safety of the surgery. Therefore, higher myopic diopter correction (or lower negative refractive error) was found to be correlated with smaller optic zone in this research (r = 0.688). It seems that the combined effect of refractive error correction and optical diameter had a greater influence on the repeatability of the Pentacam by affecting the optical quality of the postoperative corneal surface. As reported in a previous study^30^, smaller optical diameter and more myopic SE correction led to lower postoperative corneal optical quality, possibly reducing the repeatability of Pentacam post-operation. The correlation between RMS and surgerical parameters also decreased from Pos1M to Pos3M, possibly because of the effect of wound healing taking place during the follow-up period. Reports have also been published showing that loss of corneal transparency progressively decreased with time, resulting in improved accuracy of the corneal topography maps^31^. However, such a finding was not obvious in the current study and a longer follow-up period would be required to confirm it.

The ICP algorithm can provide reliable accuracy for topography matching^32^, and can quantify and correct for the effects of misalignments between successive maps. Elevation differences of corneal surface between successive measurements decreased significantly after ICP topography matching in the anterior (around 40%) and posterior (around 23%) corneal surfaces, especially in Pos1M and Pos3M. Although the elevation increased significantly post LASIK, the misalignment ratio between PreICP and PosICP remained similar for the anterior (around 0.40) and posterior surface (around 0.23) as shown in Table 2. This result confirmed that regular laser ablation did not increase the difficulty of apex detection, which could have led to increases in the weight ratio. As for the posterior surface, it is expected to be influenced by more error-inducing factors compared to the anterior surface, as discussed above, resulting in a lower misalignment ratio.

Although the difference from zero for several misalignment parameters were statistically significant, pre- and post-LASIK misalignment parameters shown in Table 4 were not large and may not be clinically relevent. The Overlap Area ratio of the corneal surface after topography matching were all above 0.975, and represented an acceptable repeatability of Pentacam both pre and post corneal refractive surgery. Rotational and translational misalignment parameters between successive measurements in different periods (Pre, Pos1M and Pos3M) were also not significant, while the overall CM increased significantly postoperatively. As CM was developed to combine the effects of individual misalignment components, it provided a more reliable assessment of the effects on reliability than considering individual misalignment components and whole data repeatability^32^.

The present study introduces a new approach to evaluating the repeatability of topography maps based on estimation and elimination of misalignment between successive maps using an ICP algorithm. The ICP algorithm has been a dominant method for registration of 3D free-form surfaces, and was introduced successfully to match topography maps in a rapid process (within 1 to 2 seconds)^32–34^.

The translational and rotational misalignments (x_0_, y_0_, z_0_, α, β, γ) of successive topography measurements were one cause of random errors. Quantified and isolated from random errors, the combined misalignment parameter combined the effects of all individual misalignment components and can be used to assess the effects of misalignment on the repeatability of corneal topography maps. The effect of misalignment on the total difference between successive measurements remained similar pre- and post-operatively. However, corneal refractive surgery decreased the repeatability of corneal topography measurements with higher myopic correction leading to lower repeatability, particularly for the anterior surface, which should be considered to improve reliability of post-surgery refractive assessment. Other factors influencing the repeatability of postoperative topography measurement include the optical diameter while wound healing may have an increasingly importance during the follow-up period of LASIK.

## Methods

### Study Participants

98 subjects (52 male and 46 female) aged between 17 and 43 years (mean age 23.39 ± 5.25 years) were recruited from myopic patients with spherical equivalent: ranging from −1.75 to −11.13 D (−5.56 ± 1.94 D) and who underwent Femtosecond laser-assisted LASIK treatment in the Refractive Surgery Department of the Eye Hospital, Wenzhou Medical University. All LASIK procedures were performed using the SCHWIND AMARIS platform (SCHWIND eye-tech-solutions, Kleinostheim, Germany). Corneal flaps were created with an LDV femtosecond laser (Ziemer Group, Port, Switzerland). Exclusion criteria included recent contact lens wear (soft contact lens within 2 weeks and rigid contact lens within 4 weeks), ocular disease, systemic disease, intraoperative or postoperative complications (e.g., free flap, reepithelialization) and retreatment. The study followed the tenets of the Declaration of Helsinki and was approved by the Scientific Committee of the Eye Hospital. Signed informed consent was obtained from the subjects after the procedures were explained to them. Patients were followed up for 3 months.

### Data Acquisition

The study parameters included refractive error (RE), K_h_ and K _v_, Q_30_, CCT, and corneal elevation data of the anterior and posterior surfaces. RE was measured with a phoroptor (RT-2100, Nidek Inc, Gamagori, Japan)and converted to spherical equivalent, SE. K_h_, K _v_, Q_30_, CCT and corneal elevation were provided by a Pentacam (OCULUS Optikgerate GmbH, Wetzlar, Germany) in different periods preoperative (Pre), postoperative 1 month (Pos1M) and 3 months (Pos3M). The Pentacam system was operated by a well-trained clinician (LFH) in a dim room in accordance with the manufacturer’s guidelines^22, 32^. Only data from the right eyes were collected and used in the analysis. Surgery parameters included optical diameter (OD), residual stromal bed thickness (RSB) and maximum ablation depth (MAD), and were recorded based on an operation plan and calculated using the ablation software of SCHWIND AMARIS.

### Repeatability Analysis

Topography analysis was carried out using the Iterative Closest Point (ICP) method. Results included the corneal misalignment parameters (translational displacements: x_0_, y_0_ and z_0_, and rotational displacements: α, β and γ), the combined misalignment parameter (CM) and the root mean square (RMS) of the difference in elevation before (PreICP-RMS) and after (PosICP-RMS) the ICP topography matching between successive topography measurements, The results also included the area of overlap between successive maps calculated as described in a previous study^32^.

Corneal misalignment parameters take the form of rigid-body transformations, where α, β and γ are the spatial extrinsic rotational components around x, y and z axes, respectively, and x_0_, y_0_ and z_0_ represent the translational components along x, y and z axes. Since using six independent misalignment components makes it challenging to have an overall impression of correlation between misalignment and data repeatability, CM was developed to combine their individual effects on data repeatability^32^. RMS should be zero if the second successive measurements matched perfectly with the first, however, this is unlikely to occur in all cases. Misalignments can only explain part of the RMS between successive maps, and the misalignment ratio calculated in the form (1 - PosICP-RMS/PreICP-RMS) was used to describe the contribution made by misalignment to the overall difference between successive maps.

### Statistical analysis

Analysis of variance (ANOVA) was carried out to compare the misalignments parameters, the inter-measurement difference of K_h_, K _v_, Q_30_ and CCT in different periods (Pre, Pos1M and Pos3M). Commercial software SPSS 20.0 (Chicago, USA) was utilized for all analyses and a two-tailed probability of P < 0.05 was considered statistically significant. The relationship between surgery parameters, SE and the RMS (PreICP and PosICP) were determined by Pearson partial correlation analyses and the Spearman linear correlation factor.
